# Using the RE-AIM framework to evaluate the implementation of scaling-up the Friendship Bench in Zimbabwe – a quantitative observational study

**DOI:** 10.1186/s12913-022-08767-9

**Published:** 2022-11-22

**Authors:** Ruth Verhey, Charmaine Chitiyo, Sandra Mboweni, Jean Turner, Gift Murombo, Andy Healey, Dixon Chibanda, Bradley H. Wagenaar, Ricardo Araya

**Affiliations:** 1 Friendship Bench, Harare, Zimbabwe; 2 Department of Community Medicine, Research Support Trust, University of Zimbabwe, Harare, Zimbabwe; 3 Women’s University in Africa (WUA), Harare, Zimbabwe; 4 Centre for Global Mental Health, King’s College, IOPPN, London, UK; 5 London School of Hygiene and Tropical Medicine, LSHTM, London, UK; 6 Department of Global Health, University of Washington, Seattle, WA USA; 7 Department of Epidemiology, University of Washington, Seattle, WA USA

**Keywords:** Mental health, common mental disorders (CMD), Friendship Bench, RE-AIM, sub-Saharan Africa, scale up, implementation evaluation, Low- and middle-income countries

## Abstract

**Background:**

This study aimed to evaluate the real-world implementation of the Friendship Bench (FB) – an evidence-based brief psychological intervention delivered by community health workers (CHWs) – three years after its implementation in three city health departments in Zimbabwe. Implementation sites were evaluated according to their current performance using the RE-AIM framework making this one of the first evaluations of a scaled-up evidence-based psychological intervention in sub-Saharan Africa (SSA).

**Methods:**

Using the RE-AIM guide (www.re-aim.org), the authors designed quantitative indicators based on existing FB implementation data. Thirty-six primary health care clinics (PHC) in Harare (*n*=28), Chitungwiza (*n*=4) and Gweru (*n*=4) were included. Among these clinics 20 were large comprehensive health care centers, 7 medium (mostly maternal and child healthcare) and 9 small clinics (basic medical care and acting as referral clinic). Existing data from these clinics, added to additionally collected data through interviews and field observations were used to investigate and compare the performance of the FB across clinics. The focus was on the RE-AIM domains of Reach, Adoption, and Implementation.

**Results:**

Small clinics achieved 34% reach, compared to large (15%) and medium clinics (9%). Adoption was high in all clinic types, ranging from 59% to 71%. Small clinics led the implementation domain with 53%, followed by medium sized clinics 43% and large clinics 40%. Small clinics performed better in all indicators and differences in performance between small and large clinics were significant. Program activity and data quality depends on ongoing support for delivering agents and buy-in from health authorities.

**Conclusion:**

The Friendship Bench program was implemented over three years transitioning from a research-based implementation program to one led locally. The Reach domain showed the largest gap across clinics where larger clinics performed poorly relative to smaller clinics and should be a target for future implementation improvements. Program data needs to be integrated into existing health information systems. Future studies should seek to optimize scale-up and sustainment strategies to maintain effective task-shared psychological interventions in SSA.

**Supplementary Information:**

The online version contains supplementary material available at 10.1186/s12913-022-08767-9.

## Background

Common mental disorders (CMD) such as depression and anxiety are a leading cause of disability worldwide, and the burden is especially high in low- and middle-income countries (LMIC) where up to 90% of those needing care have no access to it [1, 2]. Growing evidence suggests that interventions delivered by non-professionals such as community health workers (CHWs) could be used to narrow the treatment gap for CMDs [3–5].

The Friendship Bench (FB) is a brief psychological intervention delivered by CHWs which has shown effectiveness for predominantly female primary health care (PHC) clinic users through a cluster randomized controlled trial [6]. The program consists of up to 6 sessions of Problem Solving Therapy (PST) and, additionally, offers voluntary participation in community-based support group focusing on income generation. The FB program is described elsewhere [7].

The program was developed in Zimbabwe in 2006 [8] and has been scaled-up to over 36 primary health care clinics (PHC) in 2016 in response to the large treatment gap for CMD. CHWs are trained in problem solving therapy (PST) and behavioral activation (BA), which have been extensively used in low resource settings [9–11]. The FB program has been adapted to support PHC clinic users who were newly initiated on anti-retroviral therapy (ART) in Malawi [12].

The Zimbabwean CHWs are mostly elderly women who have been employed as health promoters by the respective city health authorities over 35y ago. They receive a basic salary and have continued to work past their retirement age due to economic necessity. They have been involved in the delivery of diverse public health initiatives which include treatment strategies for TB, HIV awareness, and community immunization campaigns commissioned by local health authorities.

The FB program was formally handed over to the Harare city health department in 2016 by the University of Zimbabwe (UZ) which had administered it as a research initiative. The formal hand over included the development of data collection tools aimed to integrate the FB into routine PHC services. Since 2016, the FB team has continued to play a technical supporting role albeit with reduced activities due to lack of funding. CHWs were expected to independently integrate the FB intervention into their ongoing health promotion work.

Transitioning from research to practice has unique challenges, and despite many guidelines for PHC staff, scale-up of evidence-based practices (EBPs) is slow and often encounters unique challenges that did not exist during research-based implementation [13–15]. Implementation science offers a robust methodology to evaluate the transition of EBPs from research to the real world setting by understanding what works, for whom and why [16, 17]. Implementation research frameworks can help in the development of evaluations that assess diverse factors influencing implementation of scaled-up interventions [18]. The Reach, Effectiveness, Adoption, Implementation and Maintenance (RE-AIM) framework is a widely used implementation science framework to evaluate outcomes of the process of translating research into practice [19, 20]. The RE-AIM framework has been used in diverse settings to assess real world performance of evidence-based initiatives [13, 15], it is not always used in its entirety but rather pragmatically to match the evaluation needs [15, 21].

Studying the FB program offered a unique opportunity to gain insight on an EBP which focuses on mental health on PHC level and has been implemented at a larger scale. We sought to understand in more detail which factors contributed to differences in implementation performance among 36 FB implementation sites [22].

## Methods

### Aim

The aim of this study was to evaluate the implementation of the FB program three years post scale up using the RE-AIM framework.

#### Preliminary work

To gain an overview of the FB activities at the 36 PHCs, we carried out a review of the FB data that should have been routinely collected in a data collection book at each clinic since 2016. However, we found that FB related data since the scale-up exercise started was not reliably available. Upon investigating reasons for the lack of reliable data, several factors became obvious: a) CHWs had not been trained in data collection and responsibilities were unclear; b) data was collected only from those clinics whose CHW supervisors received continual guidance from FB research team members which was only happening in Harare; c) FB data was not integrated in the routine clinic data collection efforts and therefore not prioritized.

Since reliable routine FB data was unavailable, we restructured our research approach which required a deviation from our protocol [22]. Instead of drawing from three years of data collection (2016-2019), we decided to collect fresh data in 2019, specifically focusing on the RE-AIM (Reach, Effectiveness, Adoption, Implementation, Maintenance) indicators described below. We based this decision on the assumption that we capture activity for the month prior to data collection, assuming this reflected the usual or at least minimum level of activity as no efforts had been made to increase the activity levels since the scale up in 2016.

### Design

#### Operationalization of the RE-AIM framework for this study

Using the RE-AIM guide (www.re-aim.org), we designed quantitative draft indicators based on availability of Friendship Bench implementation data such as PHC clinic user numbers, number of clinic staff, catchment area size, number of CHWs, FB program user number, data pertaining to program usage such as frequency of consultations, FB-related tools such as benches, questionnaires, notebooks, frequency of supervision meetings for CHWs and support group meetings for clients.

The research team decided on the final indicator definitions in an iterative process based on our understanding of RE-AIM framework’s definition during a 2-day-long meeting. The team consisted of experienced international and local mental health researchers who have expertise in program development, implementation, and evaluation. Three members of the research team had successfully developed and scaled up evidence-based practices (RA, DC, RV).

In total, we created 16 indicators (Table 1) covering three of the five domains (Reach, Adoption, Implementation) which are described below. It was not possible to cover all five domains of the RE-AIM framework due to the lack of reliable data as described above.

**Table 1:** List of indicators for three domains of RE-AIM model

Domain	Indicator	Formula	Source	Unit/value
Reach	R1. % of adults registered at clinic receiving SSQ	Number of SSQs done per month/ Number of adults attending clinic per month	Counted, Seen in clinic registry	%
	R2. % SSQ score >= 9 who get treatment	Number of SSQs >=9 per month/Number of people who received first session per month	Counted in LHW book	%
Effectiveness	Program Effectiveness was not specifically re-assessed in this implementation study due to a lack of reliable data.			
Adoption	A1. Does clinic have at least one functional bench?	Yes/no?	Counted	1 or 0
	A2. Does clinic have a CHW who attended a Friendship Bench training?	Yes/no?	Counted	1 or 0
	A3. Is there a CHW Supervisor appointed?	Yes/no?	Met with the person	1 or 0
	A4. Are blank SSQ forms available?	Yes/no?	Being shown the forms	1 or 0
	A5. Are at least 5 clients seen per month on the bench?	Yes/no?	Notes counted in LHWs book	1 or 0
	A6. Are FB cards available?	Yes/no?	Counted	1 or 0
	A7. Has the peer-led support group been held at the clinic in the past month?	Yes/no?	Reported by LHWs and visited if co-occurred with data collection visit	1 or 0
	A8. Is the CHW supervisor recording FB activities?	Yes/no?	Notebooks being shown	1 or 0
Implementation	*I1. Has/have the recorded CHW/s achieved fidelity per clinic?	# CHWs achieving fidelity in problem-solving therapy	Audio Recorded & Assessed by trained research staff using the FB fidelity checklist	Cut-off = 8, then 1 point (achieved) or 0 (not achieved) per recording
	I2. % days clinic open CHW seeing at least 1 patient	# days CHW seeing at least 1 patient / # days clinic open	Counted in LHWs notebook	%
	I3. CHW workload	# patients registered in the CHW book/# CHW allocated to see patients on the FB	Counted	%
	I4. % of clients seen on bench administered SSQ	# clients getting SSQ / # clients coming to bench	Counted in LHWs notebook & SSQ forms	%
	I5. % of fidelity in SSQ administration	# SSQs with no missing information per month / # SSQs conducted per month	Checked SSQ forms & counted	%
	I6. CHW retention/ turnover (or loss)	# CHWs still working at the facility / # CHWs allocated to facility in 2016	Counted	%

Based on the finalized indicators, a FB specific RE-AIM interview guide was designed with multiple versions for different interview partner groups (see Additional file Table 2) to elicit the additional information needed. We planned to confirm all statements made by stakeholders by requesting to be shown notebooks, notes, registries, filled out questionnaires or any other applicable documents.

**Table 2:** Number and distribution of clinic types in the three cities

Clinic types (Size)					
		Large	Medium	Small	**Total**
District	Harare	13	6	9	28
	Chitungwiza	4	0	0	4
	Gweru	3	1	0	4
	**Total**	20	7	9	36

#### RE-AIM indicators for FB

The following table lists all indicators and descriptive details for the three domains of the RE-AIM model chosen (Table 1).

### Setting

This study was carried out in 36 PHC clinics in three cities Harare (*n*=28), Chitungwiza (*n*=4) and Gweru (*n*=4).

All 36 PHC clinics were part of the FB scale-up process in 2016 in which CHWs were mandated to take the manualized FB training. CHWs are attached to PHC clinics which cater for the needs of communities in areas with high population density (“townships”) characterized by informal income generating activities. Depending on their size, PHC clinics serve between 20,000-80,000 people from the most socio-economically disadvantaged sectors of the population and are defined as large (poly clinics), medium (family health clinics) and small (satellite) clinics [23].

CHWs are present at all clinics and do health promotion at clinic level as well as outreach activities. CHWs are overseen by health managers (district health promoting officers - DHPOs). Clinic size defines how many CHWs (currently between 1-14 CHWs) are attached.

The group of CHWs who had prior experience with the FB program through their participation in the FB RCT [6] were assigned a peer-supervisory role in the PHC clinics they were attached to. Not all clinics had such a peer supervisor. Peer supervisors support other CHWs with referral issues, regular debriefing, and data collection.

**Fig. 1:**
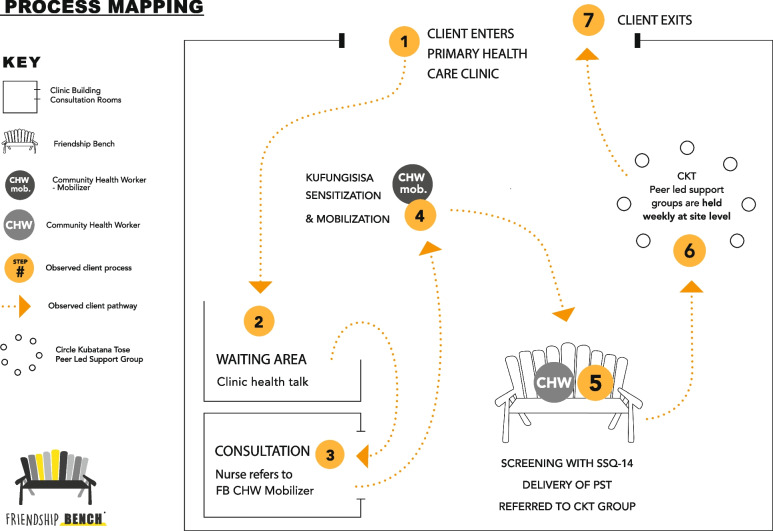
Friendship Bench patient flow in a PHC clinic. Authors RV, CC, SM, JT, DC are affiliated with Friendship Bench and therefore have permission to use the company logo

Depending on the size of the clinic, different numbers of wooden benches (Friendship Benches) are placed on the clinic premises. CHWs see clients between Monday and Thursday mornings at the clinic and at other times during the week in the informal setting of the community. Patients waiting for services at the PHC clinic are being sensitized about mental health and the FB program by FB CHWs who are trained as “mobilizers”. These can refer to the CHW on the bench who will administer a locally validated screening tool, the Shona Symptom Questionnaire (SSQ-14) [24]. Clients who score above the cut-off score and/or wish to receive the FB program are given psychoeducation and problem-solving therapy (PST). The intended FB workflow and its steps is described in the patient flow chart (Fig 1). Clients are encouraged to come back for follow-up sessions for up to 4-6 times. All clients are invited to join a peer-led support group which focuses on income generation activities such as crocheting bags out of recycled plastic or doing community gardening. Group meetings happen weekly on the clinic grounds and are facilitated by the CHWs.

### Data Collection

We investigated FB activities in 20 large, 7 medium, and 9 small clinics, as shown in Table 2.

The study was authorized by the city health authorities who run the PHCs and had received ethics approval from the Medical Research Council Zimbabwe (MRCZ). All 36 clinic leads were informed about purpose and duration of the study and key informant groups [CHWs, CHW supervisors, nurses in charge, and District Health Promotion Officers (DHPO)].

Four trained research assistants collected data between October and November 2019. Two research teams visited first clinics in Harare, then Gweru and Chitungwiza. Two consecutive days were spent at each clinic to conduct all questionnaire-based interviews and each team covered two clinics per week.

As each clinic had varying numbers of CHWs and we had time constraints, half of all FB counsellors were selected randomly in their presence and subsequently interviewed. All FB program activities for a period of one month prior to the data collection were investigated and data was entered into KOBOtool (http://support.kobotoolbox.org/) to allow for complete data collection. CHW notes and notes from the supervisor of the CHWs were read to verify the responses given in the interviews. Data was verified on site after collection and then uploaded daily onto a password protected cloud. Only the local research team had access to the data base.

### Analyses

To estimate the performance achieved at each clinic, we used descriptive and inferential statistics to analyze the quantitative data. The indicators for each domain were weighted as equivalent. All indicators were rated using a binary scale (0=not present or 1=present), except for the two indicators for Reach that were assigned % values. Indicator scores represented the level of performance, mean results per clinic group are presented.

Additionally, individual indicator scores were summed, and each clinic received a total summary score for each domain which was ranked according to a procedure suggested by Farris et al. [25]. The higher the score or % value per clinic, the further up on the ranking a clinic was, which means the better the performance in that domain.

Within each indicator and domain, there were cases where several clinics had the same combined domain rank and, therefore, ended up with the same rank.

The mean of all three domain ranks per clinic formed a composite ranking which represented the overall level of FB site performance (see Additional file Table 1). We ranked all clinics again based on this total score with the assumption that a lower score means a higher rank. Inter-Item correlations of domains were calculated.

Considering that the data was collected from all clinics participating in this study and that there was homogeneity of variance across the clinics, an analysis of variance (ANOVA) was used to determine any significant differences among the clinics’ scores and differences according to type of clinic. Additionally, a Tukey post hoc test was used to confirm the differences between clinics individually and when aggregated as type groups.

To establish whether clinic types had an influence on the performance of a FB site, we aggregated the clinics according to their types (large, medium sized, small) and compared their performance using their overall composite ranking (mean of all 3 ranks).

## Results

Our questionnaires were administered to 152 key stakeholders: Community Health workers (*n*=74), Community Health Worker supervisors (*n*=35), nurses in charge (*n*=36) and the district health promoting officers (DHPOs) (*n*=7). Only one approached key informant, a District Health Promotion Officer (DHPO) from Harare, was not available for the interview.

### Final performance of clinics based on RE-AIM outcomes

Each clinic had a result for each indicator in all three domains. Table 3 shows the results for the three clinic types. Table 3 shows the average score in percentages for each of the REAIM domains by clinic size. Small clinics had the highest cumulative reach of 33.78% (SD 15.42), while medium clinics had the smallest reach of 9.39% (SD 11.34). In keeping with this trend, small clinics had the highest adoption average score of 70.83 (SD 12.50), while medium clinics had the lowest average adoption score of 58.93% (SD 24.7). Under implementation, large clinics had the lowest average score of 39.54% (SD 17.66), while small clinics maintained the trend of having the highest average score, with an implementation average score of 53.29% (SD 10.23).

**Table 3:** Results of clinic size groups by domain (means, SD)

	Small ***n***=9	Medium ***n***=7	Large ***n***=20
**Reach (in %)**	33.8 (SD 15.4)	9.4 (SD 11.3)	15.3 (SD 19.2)
**Adoption (in %)**	70.8 (SD 12.5)	58.9 (SD 24.7)	60.6 (SD 17.3)
**Implementation (in %)**	53.3 (SD 10.2)	43.1 (SD 24.5)	39.5 (SD 17.7)

All values are percentages

Based on individual clinics’ scores per domain, all clinics were ranked for the size group (Table 3). Fourteen clinics had the same rank average and therefore the same final rank (appendix 1). There was substantial variability in the rankings across domains for each clinic. For instance, the best overall ranked clinic was best ranked for implementation but came only 13^th^ for reach. A similar pattern could be observed for the next 6-8 top ranked clinics. As shown in Table 4, among lower performing clinics there was more consistency in the ranks across all domains. Similarly, large clinics were overrepresented in the group of low performing clinics. Ranks across domains were more consistent for the predominantly small and medium sized clinics in the high performing clinic group.

**Table 4:** Correlations between the three domains (Reach, Adoption, and Implementation)

	Final Rank	Reach Rank	Adoption Rank	Implementation Rank
**Final Rank**	1.000			
**Reach Rank**	.746 (*p*=0.000)	1.000		
**Adoption Rank**	.602 (*p*=0.000)	.177 (*p*=.300)	1.000	
**Implementation** **Rank**	.710 (*p*=0.000)	.369 (*p*=.027)	.281 (*p*=.096)	1.000

Final rank scores ranged from 1-36. From this distribution of mean ranks across the three types of clinics, Levene's test indicated homogeneity of variance [*F*(2, 33) = 1.08 *p* = .352]. Therefore, the distribution and/or spread of scores around the mean of the three clinic types were considered equal. Analysis of variance showed a significant difference between the types of clinics based on their final ranking [*F*(2, 33) = 6.79, *p* = .003].

Post hoc analyses using Tukey’s HSD (Tukey honestly significant difference) indicated that rankings were significantly lower for larger than smaller clinics (*p* = .002), rankings did not differ significantly between large and medium clinics (*p* = .38) as well as between those of small and medium size (*p* = .20).

### Relationship among domains

Looking at the correlations among domain ranks, the relationships were found to be small and not significant (*p*<0.05) confirming the independence of domains in terms of classifying levels of performance. As expected, the ranks for all domains correlated strongly with the total ranks. Nonetheless, small differences can be observed. The Adoption domain ranking results had marginally lower correlation with total ranks (*r* = .60) compared to the reach (*r* = .75) and implementation (*r* = .71) ranking results (see Table 4).

## Discussion

To our knowledge, this is the first study that uses the RE-AIM framework to evaluate the performance of a mental health program three years after being scaled up in Sub-Saharan Africa. Studies that have used the RE-AIM framework have been largely based in the Northern hemisphere. FB clinics were classified according to their performance as part of an effort to understand contextual factors influencing the implementation of the FB program three years post-scale up [22]. Our results were designed to be used together with our other study focusing on key stakeholder perspectives (still unpublished) [22] to help develop specific strategies to address implementation barriers [26, 27].

Context can be understood as all aspects that are not the intervention [28] and contextual determinants such as clinic level characteristics have been found to have an influence on clinical outcomes [29].

We discovered that clinic size influenced performance of the FB program. Overall across our evaluation, small clinics, albeit existent only in the capital Harare, showed stronger FB implementation compared to large clinics. This association was particularly strong for the domain Reach, with small clinics achieving almost 4-times the reach of medium clinics (34% vs. 9%). The reason for this could be that CHWs in small clinics have more time to focus on the FB program and mobilize those coming to the clinic. Community members receive care in small clinics before being referred on. Thus, their first contact with FB is in small clinics and they will not reengage with the program once referred to a large clinic (unpublished data). Patients reported not having time to speak to FB CHWs at large clinics when seeking care for specific health issues (unpublished data). Small clinics also had better rates of Adoption and Implementation domains. For example, were the wooden benches that are used by CHWs and their clients more in order due to less patient traffic. CHWs at small clinics deliver fewer competing programs which enables them to focus more fully on the FB program.

Admittedly, the FB program had been discontinued in both other cities (Gweru and Chitungwiza) as health authorities had reduced their focus on mental health care and redirected CHWs to other duties (unpublished data). Despite the program being inactive, we collected data on various domain indicators which placed clinics in the ranks showing weak performing. From a systems perspective, successful program implementation depends on all combined program factors explored in this study and hypothetically barriers on one area affect other domains but this study shows that domains performed rather independently among high performance clinics but there was much stronger correlation across domains at the bottom of the overall performance ranking.

Additionally, parallel research carried out by the FB research team led to more contact and support for CHWs based in clinics located in Harare as well as collaboration with the city authority staff which led to the FB program being continuously offered between 2016 and 2019.

Harare based small clinic settings with less competing health care program and ongoing support from the program designers as well as from the health authorities had better FB program performance compared to bigger clinics.

Furthermore, we had learned in the formative phase that the FB related data collection was not integrated into the clinic data reporting system as it was not a priority in the clinics’ activities. Health information systems in LMIC are often affected by lack of reliable data [30]. Our finding highlights the importance of well-structured health information systems that are aligned with users and settings, which lay out and support care pathways and prioritize data collection to support person-centered and evidence-informed care [31]. Low data quality is a common problem in the process of transferring responsibility from a research project to organizations implementing an EPB [32, 33] and needs to be addressed when strengthening the under-resourced mental health care systems in LMIC [34, 35].

An additional important outcome of this study was discovering barriers that hindered the FB program which we decided to address right after we finished data collection. We reengaged and supported the City Health departments and clinic leads in all three cities by distributing material, repairing or replacing benches, and offering refresher trainings to the CHWs to ensure a new start and/or continuation of the FB program, respectively.

Scaling up efforts need to be well planned and strongly supported to prevent losing fidelity of the program and data should be used to monitor the process to detect and overcome difficulties as soon as it may be possible [4].

Scaling a program like the FB requires an approach that takes into account contextual determinants such as clinic setting, availability of program specific aspects, structural support from all stakeholders and emphasizes the integration of the program data into the existing health information system. The data collection process has to be designed in a “user-friendly” way when delivering agents are being relied on to document and report program activities.

This study has contributed to our understanding of the implementation, these results and the ones of the follow up study (unpublished data), we create strategies to address barriers and enablers (unpublished data) as laid out in the study protocol [22].

### Limitations

Only clinics in Harare, the capital of Zimbabwe, still had program activity at the point of data collection which makes the results less generalizable. As scale up data since 2016 was insufficient and unreliable, we had to focus on the implementation activities one month before our research visits which does not reflect well the three years prior of FB program activities.

Some RE-AIM domains were not assessed and the need to weight indicators and outcomes for the domains included is debatable. As routine data collected prior to the study was unreliable, we had to choose indicators according to what our expert team agreed on and what was possible to obtain. Since the study, we implemented ways to ensure better data collection such as hiring data clerks in each city, distribution of tablet computers and use of a data collection app that was created inhouse. Additionally, we have put in place a financial reward system for all FB CHWs for correct data reporting.

Unfortunately, we only had a limited time at each implementation site to carry out data collection and some results of observations might therefore be less reliable. For instance, there was relatively little patient traffic during the observation period which also affected our plan to measure fidelity through analysis of audio recordings which we were only able to collect in insufficient numbers and had to leave out of this analysis.

Despite having a large key informant sample, we were not able to interview all of them due to logistic constraints. However, we think the sample interviewed is representative of the staff working in the FB.

## Conclusions

Overall, our RE-AIM evaluation found strong ongoing implementation and adoption of the Friendship Bench program over 3 years after implementation was passed to local City Health officials. However, Reach across all clinics was relatively low and especially low for large clinics. Across all RE-AIM domains, small clinics performed better than large clinics. Future studies should examine heterogeneity in RE-AIM indicators across key contextual factors in low-resource settings, as well as seek to optimize scale-up and sustainment strategies to maintain effective program implementation over the long term.

## Supplementary Information

**Additional file 1: Table 1.** Overall ranking for all clinics (*n*=26).

**Additional file 2: Table 2**. OPT FB RE-AIM Main Study Questionnaire 2019.

## Data Availability

The dataset generated and analyzed during this study is available from the Friendship Bench data repository upon reasonable request from the corresponding author Ruth Verhey, email: ruth.verhey@zol.co.zw.

## Abbreviations

CHW: Community health worker
CHW S: Community health worker supervisor
DHPO: District health promoting officer
FB: Friendship Bench
FHS: Family health service
OI: Opportunistic infections
PHC: Primary health care
RE-AIM: Reach Effectiveness Adoption Implementation Maintenance
SSQ: Shona Symptom Questionnaire
Tukey’s HSD: Tukey honestly significant difference
