# Supplementary material for: Using the RE-AIM framework to evaluate the implementation of scaling-up the Friendship Bench in Zimbabwe – a quantitative observational study
Source: BMC Health Serv Res. 2022 Nov 22;22:1392. doi: 10.1186/s12913-022-08767-9 (PMC9682765; doi:10.1186/s12913-022-08767-9)
Supplement: Supplementary file 1 — Additional file 1: Table 1. Overall ranking for all clinics (n=26). [file 12913_2022_8767_MOESM1_ESM.docx]

Additional file table 1 is showing the overall ranking for all individually listed clinics showing clinic type.

<insert additional file table 1>

Additional file table 1: Overall ranking for all clinics (n=26)

| Clinic Name | Clinic Type | Combined Reach Rank | Combined Adoption Rank | Combined Implementation Rank | Final Rank |
| --- | --- | --- | --- | --- | --- |
| A | Small | 13 | 4 | 1 | 1 |
| B | Small | 1 | 4 | 16 | 2 |
| C | Large | 3 | 1 | 18 | 3 |
| D | Medium | 4 | 16 | 5 | 4 |
| E | Small | 2 | 4 | 22 | 5 |
| F | Small | 4 | 2 | 23 | 6 |
| G | Medium | 13 | 16 | 1 | 7 |
| H | Large | 24 | 2 | 6 | 8 |
| I | Small | 9 | 16 | 8 | 9 |
| J | Small | 18 | 8 | 10 | 10 |
| K | Large | 19 | 4 | 14 | 11 |
| L | Large | 7 | 16 | 15 | 12 |
| M | Small | 11 | 16 | 12 | 13 |
| N | Medium | 23 | 8 | 8 | 13 |
| O | Medium | 26 | 11 | 3 | 15 |
| P | Large | 15 | 11 | 17 | 16 |
| Q | Small | 8 | 16 | 20 | 17 |
| R | Large | 22 | 16 | 6 | 17 |
| S | Large | 15 | 26 | 4 | 19 |
| T | Medium | 9 | 26 | 11 | 20 |
| U | Small | 4 | 26 | 21 | 21 |
| V | Large | 11 | 16 | 24 | 21 |
| W | Large | 25 | 8 | 18 | 21 |
| X | Large | 17 | 11 | 33 | 24 |
| Y | Large | 19 | 32 | 12 | 25 |
| Z | Large | 28 | 11 | 27 | 26 |
| AB | Medium | 28 | 11 | 27 | 26 |
| AC | Large | 21 | 26 | 27 | 28 |
| AD | Large | 27 | 26 | 25 | 29 |
| AE | Large | 28 | 16 | 34 | 29 |
| AF | Large | 28 | 16 | 35 | 31 |
| AG | Large | 28 | 26 | 27 | 32 |
| AH | Large | 28 | 32 | 26 | 33 |
| AI | Large | 28 | 32 | 27 | 34 |
| AJ | Large | 28 | 32 | 27 | 34 |
| AK | Medium | 28 | 36 | 36 | 36 |
