# Supplementary material for: Using the RE-AIM framework to evaluate the implementation of scaling-up the Friendship Bench in Zimbabwe – a quantitative observational study
Source: BMC Health Serv Res. 2022 Nov 22;22:1392. doi: 10.1186/s12913-022-08767-9 (PMC9682765; doi:10.1186/s12913-022-08767-9)
Supplement: Supplementary file 2 — Additional file 2: Table 2. OPT FB RE-AIM Main Study Questionnaire 2019. [file 12913_2022_8767_MOESM2_ESM.docx]

Additional file table 2: OPT FB RE-AIM Main Study Questionnaire 2019

**1 Clinic Profile**

| **Clinic Name** | **Clinic Type** | **Date** | **Day of Field Visit (1/2)** | **RA Name** | **RA ID** | **Respondent**  **LHW/LHWs**  **/Nurse/DHPO** |
| --- | --- | --- | --- | --- | --- | --- |
|  |  |  |  |  |  |  |
|  |  |  |  |  |  |  |

RE-AIM (Reach, Effectiveness, Adoption, Implementation and Maintenance)

(***Ask respondent to show the source document with information on the specific question***)

**2 REACH**

| **Question** | **Response** |
| --- | --- |
| 1. **Number of adults registered at clinic**   1 How many adults have come to the clinic in the past 30 days?  ***(ask respondent to show you the source of information)***  ***2Is information source available?*** |  |
| **B Adults registered at clinic receiving SSQ**  1 How many people were screened with the SSQ in the past 30 days?  **(ask respondent to show you the source of information)**  2 Are you administrating the SSQ questionnaire to all your clients?  3 Do you have SSQ form to confirm the numbers?  4 If yes how many are available for the past 30 days?  **(ask to see the SSQ forms)**  5 If No, where are the scores recorded? **(*ask respondent to show you were SSQs are recorded*)**  6 Is source available | **1 (Record Total)**  **1 Yes to all clients**  **2 to the majority of my clients not all of them**  **3 No I am not using the SSQ at all**  **1Yes**  **2No**  ***1 (Record Total)***  **1CHW Book**  **2 CHWS Book**  **3 Nurse’s File**  **4 Patients card**  **5 Source but numbers do not match?**  **6 No source**  **7Don’t know**  **1 Yes**  **2 No** |
| **C Adults with SSQ >=9**  1 How many people have scored >= 9 in SSQ in the past 30 days?  ***(ask respondent to show you source of SSQ scores)***  *2 Is source of information available?* | **(Record *total number***)  1 Yes  2 No |
| **D Adults referred after red flag**  *1* How many got red flags in the past 30 days?  2 How many of these did you refer?  (***Ask respondent to show you the source of red flags)***  3 Is source of information available  4 Where/who did you refer to? | ***(Record total number*)**  ***(Record total number)***  **1 Yes**  **2 No**   1. **Nurse** 2. **DHPO** 3. **Private doctor** 4. **Psychiatric Unit Harare Central Psychiatric Unit** 5. **Parirenyatwa Hospital** |
| **E Adults receiving FB intervention (Filled out FB card or client reported in CHW book)**  1 How many people got a first session on the bench in the past 30 days?  ***(Ask respondent to show you: CHW book/CHW S book/FB card)***  *2 Is source of information available*  3 How many people got a follow up session on the bench in the past 30 days?  ***(Ask respondent to show you: CHW book/CHW S book/ FB card)***  *4 1 Is source available* | ***(Record total)***  ***1 yes***  ***2 No***  ***(Record total)***  **1 Yes**  **2 No** |
| **F Adults receiving 4 sessions or more (incl. Informal sessions outside of clinic, home visits)**  1 How many people were seen for 4 or more sessions in the past 30 days)  ***(Ask respondent to show you: CHW book/ CHW S book/ FB card)***    2 Is source of information available | **(Record total)**  **1 Yes**  **2 No** |
| 1 In the past 30 days, on how many days were clients seen on Friendship Bench in your clinic, receiving any sessions  **(ask respondent to show you source)** | **Record days** |

**3 Adoption**

*For all physical items that the respondent says are available at the clinic, please ask to physically see them.*

| 1. How many CHWs work in this clinic? | **Record Total** |
| --- | --- |
| 1. How many are working on the Friendship Bench? | **Record Total** |
| 1. How many are FB mobilizers? | **Record Total** |
| 1. How many do both work on the bench and mobilize? | **Record Total** |
| 1. How many usable Benches does this clinic have? | **Record Total** |
| 1. How many benches are not usable (broken)? | **Record Total** |
| 1. Is there someone responsible for FB Bench Maintenance?   7a. If yes who is responsible for the maintenance (title of person)? | **1Yes**  **2No**  **1 CHW**  **2 General Hand**  **3 Grounds man** |
| 1. How many CHWs have gone through the FB training | **Record Total** |
| 1. How many CHWs have gone through the FB training | **Record Total** |
| 1. Does this clinic have a CHW supervisor? | **Yes**  **No** |
| 1. Do you have blank SSQ forms available? | **Yes**  **No**  **Have one left** |
| 1. Who uses the SSQ form/s? | **1 CHW**  **2 CHWS**  **3 Nurse**  **4 DHPO**  **5 Don’t know** |
| 1. Do you have blank PHQ forms available?   **(ask respondent to show you)** | **1 Yes**  **2 No**  **3 Don’t Know**  **4 We have one left** |
| 1. Who uses the PHQ form/s? | **1 CHW**  **2 CHWS**  **3 Nurse**  **4 DHPO**  **5 Don’t know** |
| 1. Do you have *blank?* (CHW) books available?   **(ask respondent to show you LHWs Book)** | **Yes**  **No** |
| 1. Who uses the CHW Book? | **1 CHW**  **2 CHWS**  **3 Nurse**  **4 DHPO**  **5 Don’t know** |
| 1. Do you have blank FB cards available?   **(ask respondent to show FB cards)** | **1 Yes**  **2 No**  **3 We have one left** |
| 1. Who uses the FB Card? | **1 CHW**  **2 CHWS**  **3 Nurse**  **4 DHPO**  **5 Don’t know** |
| 1. Do you have blank LHW supervisor books available? | **1Yes**  **2 No** |
| 1. How many LHWs have seen at least 5 clients on the bench (no informal sessions) in the last 30 days   (ask respondent to show you source) | **Record Total** |
| 1. Is there a DHPO assigned to your clinic?   *21a. If yes, what is the name of the DHPO* | **1 Yes**  **2 No**  **3 Don’t know**  **Record Name** |
| 1. Do you have sensitization talks at this clinic?   22a. If yes, how many are done per day?  22b. Where are they done? | **1 Yes**  **2 No**  **3 Don’t know**  **Record total**  **1At the gate**  **2 At the waiting**  **room for OI clinic**  **3 At the waiting**  **room for**  **maternity clinic**  **4 At the waiting**  **room for FHS**  **5No sensitization**  **Talks** |
| 1. Does your clinic have a CKT (circle kubatana tose?)   23a. If yes how many times has CKT happened in the past 30 days? | **1 Yes**  **2 No**  **3 Don’t Know**  **(Record total)** |
| 1. (If clinic is a poly clinic) Have health talks taken place at the clinic 2. If yes where do the health talks take place? 3. Who gives the health talk? 4. If FB mentioned in the health talk? | **1 Yes**  **2 No**  **1 FHS**  **2 Maternity**  **3 OI**  **4 All three departments**  **1 Clinical Nurse**  **2 CHW**  **3 CHWS**  **1 Yes**  **2 No** |
